# Supplementary material for: Characterization of Bacterial Transcriptional Regulatory Networks in Escherichia coli through Genome-Wide In Vitro Run-Off Transcription/RNA-seq (ROSE)
Source: Microorganisms. 2023 May 25;11(6):1388. doi: 10.3390/microorganisms11061388 (PMC10301672; doi:10.3390/microorganisms11061388)
Supplement: Supplementary file 1 [file microorganisms-11-01388-s001.zip › microorganisms-2390527-supplementary.pdf]

Supplementary data for Characterization of Bacterial Transcriptional Regulatory Networks in *Escherichia coli* through Genome-Wide in vitro Run-Off Transcription/RNA-seq (ROSE)

Table S1. Technical comparison between the two genome-wide *in vitro* transcriptome sequencing techniques ROSE and RIViT-seq [9].

	ROSE	RIViT-seq
**Isolation of genomic DNA**	- Three different isolation methods tested (Quick-DNA Universal Kit by Zymo, NucleoSpin Microbial DNA Kit by Macherey Nagel and Phenol-Chloroform Isoamyl alcohol DNA extraction)	- GenElute Bacterial Genomic DNA kit (MilliporeSigma)
**Digestion of genomic DNA**	- Random fragmentation (average size 6 kb) using gTubes (Covaris)	- Enzymatic digestion with *Eco*RI, *Hind*III, *Bam*HI and *Xho*I
**RNA purification after *in vitro* transcription**	- RNeasy MinElute Kit (Qiagen)	- RNeasy MinElute Kit (Qiagen)
**Primary transcript library generation**	- 100 ng RNA input<br>- Fragmented to an average size of 500 nt<br>- Digestion of transcripts with 5′ di- and monophosphates by terminator exonuclease (Epicentre)<br>- Ligation of RNA index adapters<br>- Treatment with RNA 5′-polyphosphatase (New England Biolabs)<br>- Ligation of RNA adapters<br>- Reverse-transcription to cDNA using a sequence-independent loop adapter<br>- Amplification of cDNA with barcoded primers	- 6 µg RNA input<br>- /<br>- /<br>- /<br>- Treatment with RNA 5′-polyphosphatase (New England Biolabs)<br>- Ligation of RNA adapters<br>- Reverse-transcription to cDNA using a random hexamer with an adaptor sequence<br>- Amplification of cDNA with barcoded primers
**Whole-genome transcriptomics**	- /	- Stranded cDNA library generation using TrueSeq Stranded RNA Library Prep Kit (Illumina) and low sample protocol
**Sequencing and data processing**	- High throughput single-end sequencing (1x75bp)<br>- Illumina MiSeq<br>- Trimming using Trimmomatic<br>- Mapping with Bowtie2	- High throughput paired-end sequencing (2x150bp)<br>- Illumina MiSeq<br>- Trimming using BBDuk<br>- Mapping with HISAT2
**Sequence analysis**	- TSS detection and visualization with ReadXplorer, Improbizer, and MEME/Weblogo	- TSS detection manually with read counts of samtools, motif visualization with MEME<br>- Differential Expression analysis with DESeq2

Table S2. Mapping statistics for all six 5′-end specific ROSE- Eσ^{70} libraries. Mapping statistics for ROSE- Eσ^{70} libraries with the isolation methods by Zymo Research (Z1; Z2), Macherey-Nagel (M1; M2) and with Phenol-Chloroform Isoamyl alcohol (P1; P2).

	**Z1**	**Z2**	**M1**	**M2**	**P1**	**P2**
**Mappings**	2,061,241	2,086,867	1,618,341	2,076,393	2,210,864	1,737,821
**Unique Mappings**	2,061,241	2,086,867	1,618,341	2,076,393	2,210,864	1,737,821
**Single Perfect Mappings**	1,572,385	1,553,410	1.240,224	1,533,963	1,680,056	1,305,272

Table S3. Mapping statistics for four 5′-end specific *in vivo* libraries. Mapping statistics for *in vivo* libraries of the wildtype strain (WT), the *Δfur* knockout strain (*Δfur*), the *Δfis* knockout strain (*Δfis*) and of the *Δhns* knockout strain (*Δhns*).

	**WT**	***Δfur***	***Δfis***	***Δhns***
**Mappings**	1,605,710	928,706	493,254	597,949
**Unique Mappings**	1,605,710	928,706	493,254	597,949
**Single Perfect Mappings**	1,288,944	760,574	344,796	438,708

Table S4. Transcription start site detection parameters for ROSE-Eσ^{70} libraries in ReadXplorer [13,17].

**Parameter**	**Value**
Minimum number of read starts:	7
Minimum percent of coverage increase:	148
Maximum low coverage read start count:	0
Minimum low coverage read starts:	0
Detect novel transcripts?	Yes
Minimum transcript extension coverage:	-
Maximum distance to feature of leaderless transcripts:	300
Associate nearby neighboring TSS?	Yes
Associate neighboring TSS in a bp window of:	3
Minimum mapping quality:	0
Single Perfect Match included:	Yes
Perfect Match included:	No
Single Best Match included:	Yes
Best Match included:	No
Common Match included:	No
Include multiple mapped reads:	Yes
Mapping strand selection:	Feature/analysis strand

Table S5. Transcription start site detection parameters for *in vivo* libraries in ReadXplorer [13,17].

**Parameter**	**Value**
Minimum number of read starts:	1
Minimum percent of coverage increase:	32
Maximum low coverage read start count:	0
Minimum low coverage read starts:	0
Detect novel transcripts?	Yes
Minimum transcript extension coverage:	20
Maximum distance to feature of leaderless transcripts:	300

Associate nearby neighboring TSS?	Yes
Associate neighboring TSS in a bp window of:	3
Minimum mapping quality:	0
Single Perfect Match included:	Yes
Perfect Match included:	No
Single Best Match included:	Yes
Best Match included:	No
Common Match included:	No
Include multiple mapped reads:	Yes
Mapping strand selection:	Feature/analysis strand

Table S6. New potential promoter regions repressed by Fur-Fe^{2+}, Fis and HN-S discovered by ROSE-$E\sigma^{70}$.

Transcription factor	Position of detected transcription start site	Potential repressed gene
**Fur-Fe^{2+}**	87,999 bp	*cra*
	297,045 bp	*intF*
	2,037,669 bp	*yedV*
	3,313,170 bp	*rbfA*
**Fis**	257,943 bp	*crl*
	297,045 bp	*intF*
	953,680 bp	*pflB*
	1,010,924 bp	*uup*
	1,900,554 bp	*yoaE*
	3,371,426 bp	*nanT*
	3,384,275 bp	*mdh*
	3,805,312 bp	*waaS*
	4,561,366 bp	*yjiH*
	4,612,302 bp	*rsmC*
**H-NS**	297,045 bp	*intF*
	1,804,195 bp	*thrS*
	1,946,226 bp	*yebS*

	2,481,794 bp	*evgA*
	2,774,364 bp	*yfjV*
	3,154,942 bp	*yqhC*
	3,227,445 bp	*ygjH*
	4,327,049 bp	*crfC*
	4,556,211 bp	*yjiC*

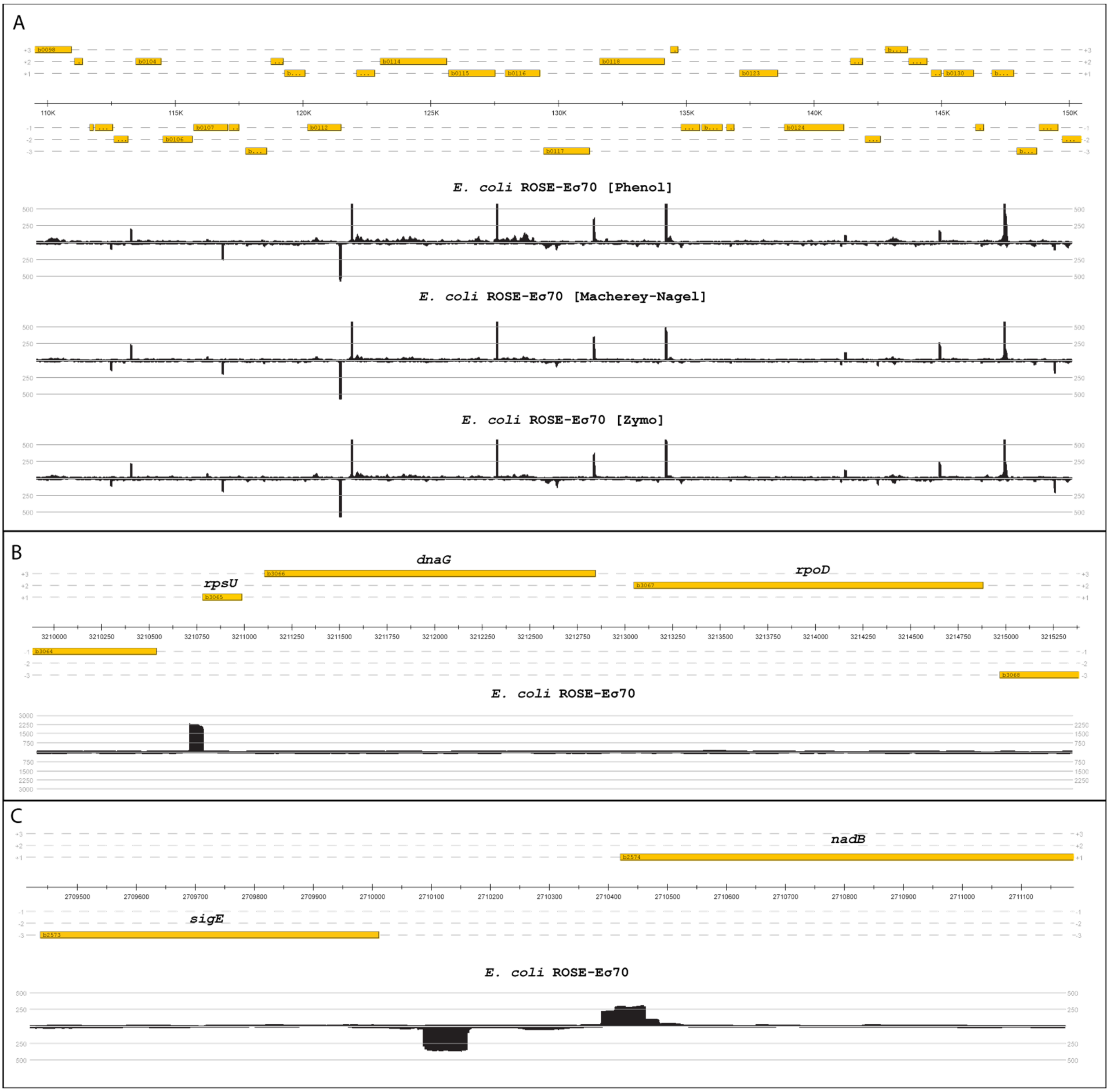

Figure S1. Mapped reads of the 5′-end-specific transcript library in exemplary genomic regions. The reads (black) are filtered and mapped to the reference genome U00096.3. The coverage of the reads associated to their respective location on the genome is visualized with ReadXplorer [13]. (A) Representation of the mapped read distribution in the genomic region 110,000-150,000 bp of the *E. coli* genome. The genomic DNA for the ROSE-Eσ^{70} experiment was isolated with Phenol-Chloroform Isoamyl alcohol (top), with the NucleoSpin Microbial DNA Kit from Macherey-Nagel (middle) and with the Quick-DNA Universal Kit from Zymo (bottom). (B) Exemplary read stack for the *rpsU-dnaG-rpoD* operon generated by ROSE-Eσ^{70}. (C) Exemplary read stacks for the genes *nadB* on the sense strand and *sigE* on the antisense strand generated by ROSE-Eσ^{70}.

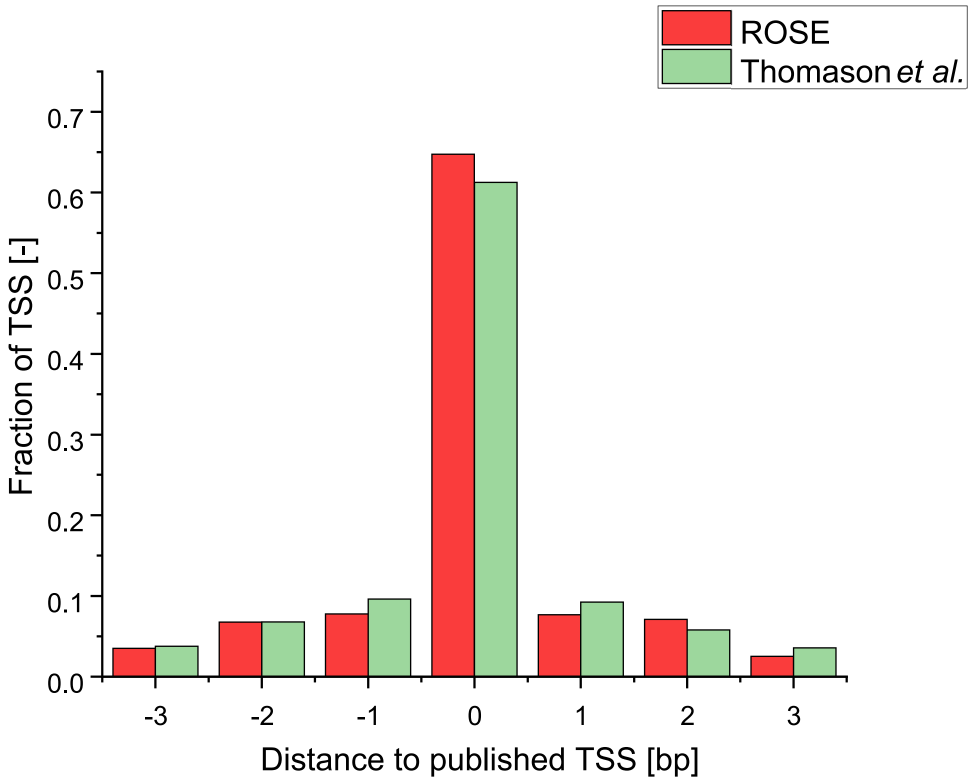

Figure S2. Distribution of the identified transcription start sites (TSS) by ROSE and by Thomason et al. [20] in relation to their distance to the published TSS in RegulonDB [22].

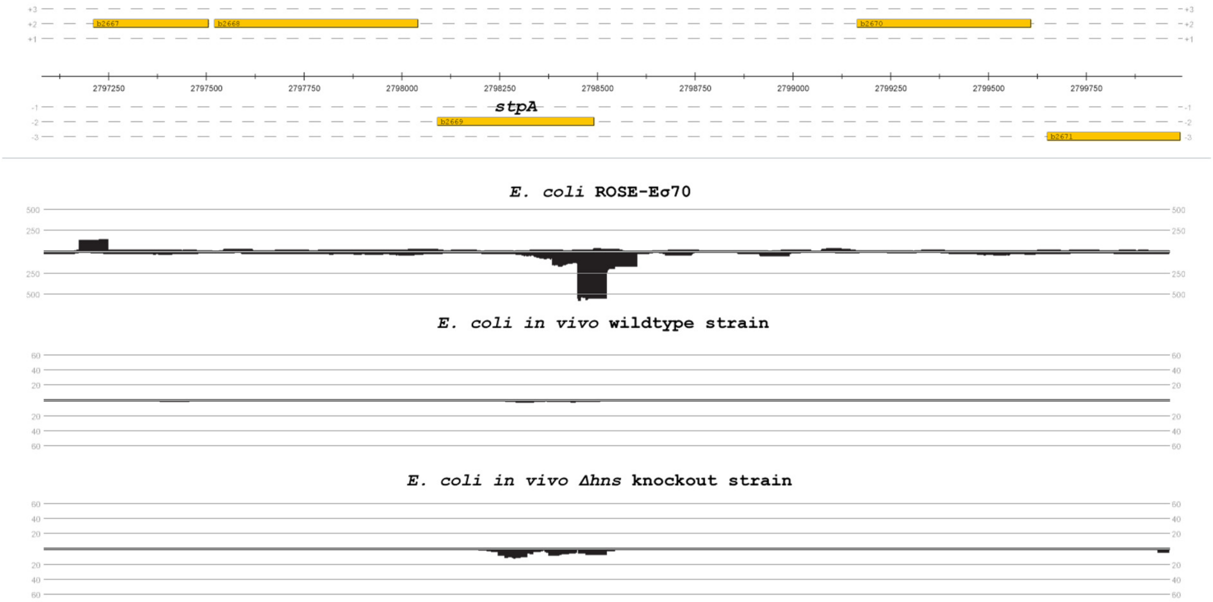

Figure S3. Coverage of mapped reads on the reference genome with an emphasis on gene *stpA*. Readcount in the promoter region of *stpA* from the *E. coli* ROSE-Eσ^{70} (top), *E. coli in vivo* Wildtype strain (middle) and *E. coli in vivo* Δ*hns* knockout strain (bottom). The mapping took place on the respective reference genome (U00096.3) and is visualized with ReadXplorer [13].

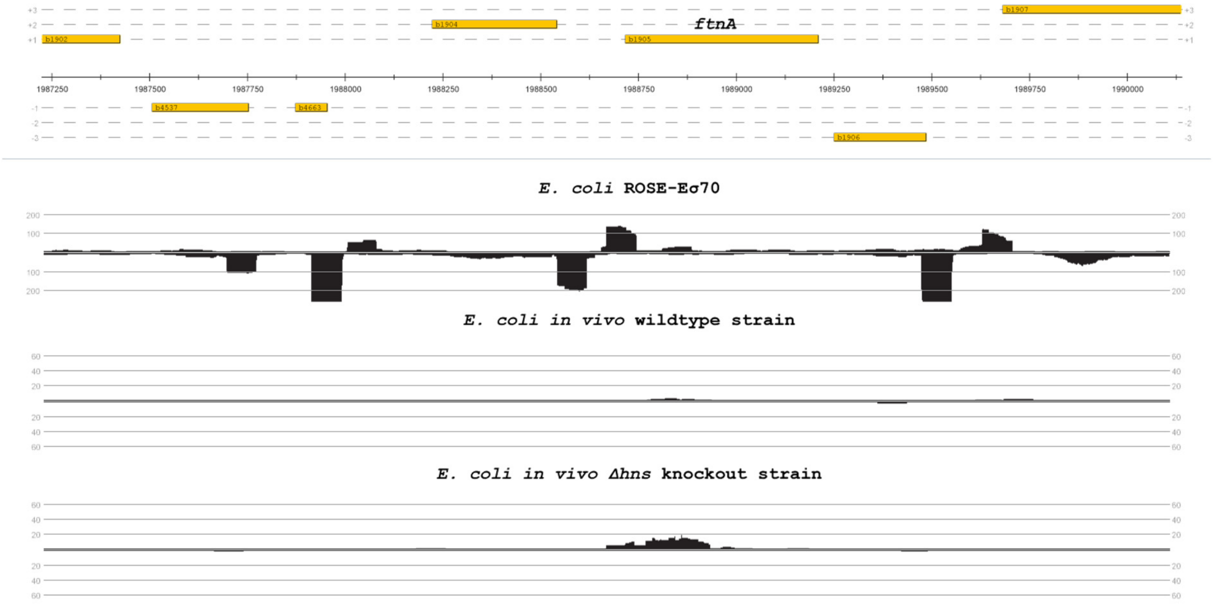

Figure S4. Coverage of mapped reads on the reference genome with an emphasis on gene *ftnA*. Readcount in the promoter region of *ftnA* from the *E. coli* ROSE-Eσ^{70} (top), *E. coli in vivo* Wildtype strain (middle) and *E. coli in vivo* Δ*hns* knockout strain (bottom). The mapping took place on the respective reference genome (U00096.3) and is visualized with ReadXplorer [13].

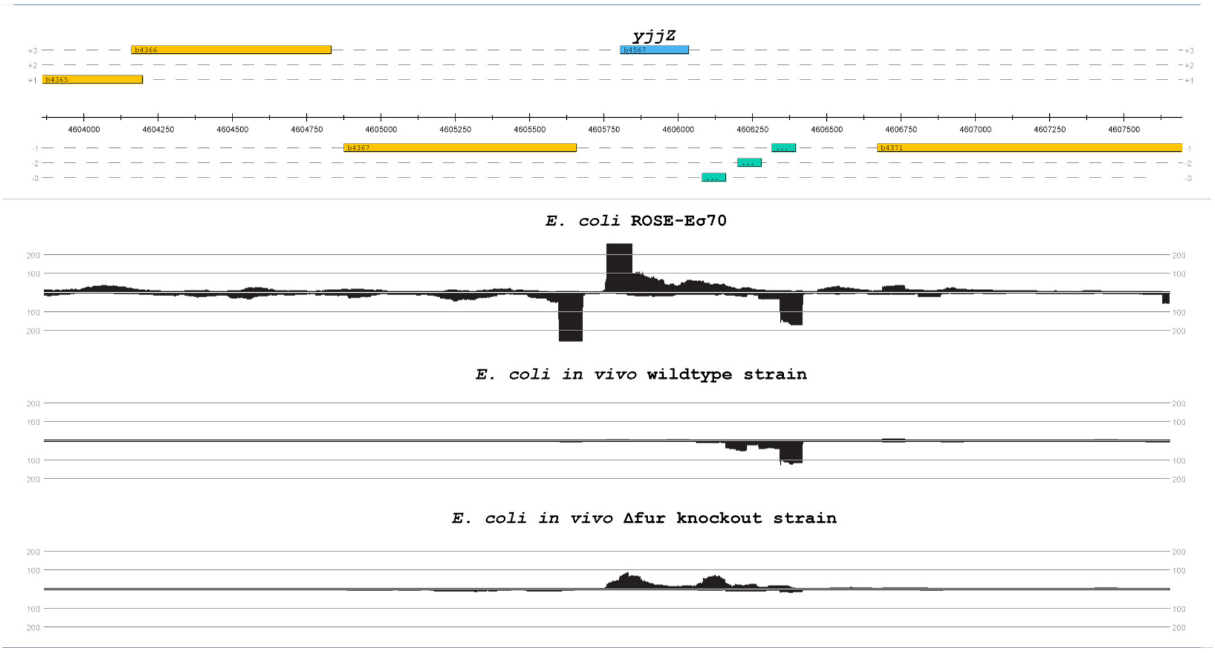

Figure S5. Coverage of mapped reads on the reference genome with an emphasis on gene *yjjZ*. Readcount in the promoter region of *yjjZ* from the *E. coli* ROSE-Eσ^{70} (top), *E. coli in vivo* Wildtype strain (middle) and *E. coli in vivo* Δ*fur* knockout strain (bottom). The mapping took place on the respective reference genome (U00096.3) and is visualized with ReadXplorer [13].

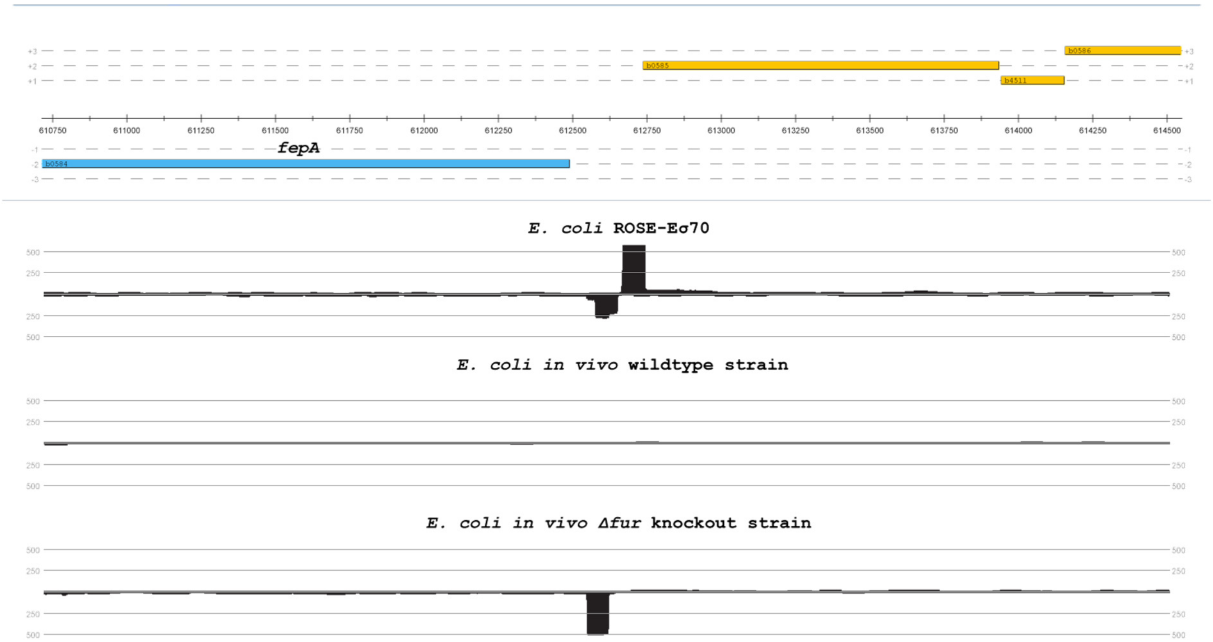

Figure S6. Coverage of mapped reads on the reference genome with an emphasis on gene *fepA*. Readcount in the promoter region of *fepA* from the *E. coli* ROSE-Eσ^{70} (top), *E. coli in vivo* Wildtype strain (middle) and *E. coli in vivo* Δ*fur* knockout strain (bottom). The mapping took place on the respective reference genome (U00096.3) and is visualized with ReadXplorer [13].

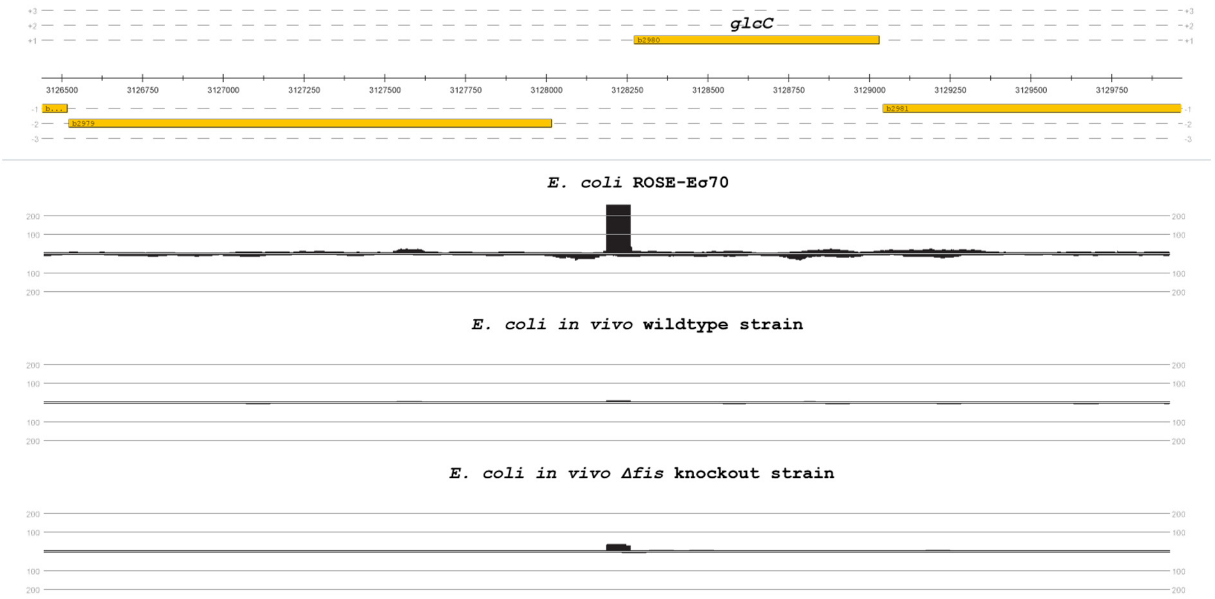

Figure S7. Coverage of mapped reads on the reference genome with an emphasis on gene *glcC*. Readcount in the promoter region of *glcC* from the *E. coli* ROSE-Eσ^{70} (top), *E. coli in vivo* Wildtype strain (middle) and *E. coli in vivo* Δ*fis* knockout strain (bottom). The mapping took place on the respective reference genome (U00096.3) and is visualized with ReadXplorer [13].

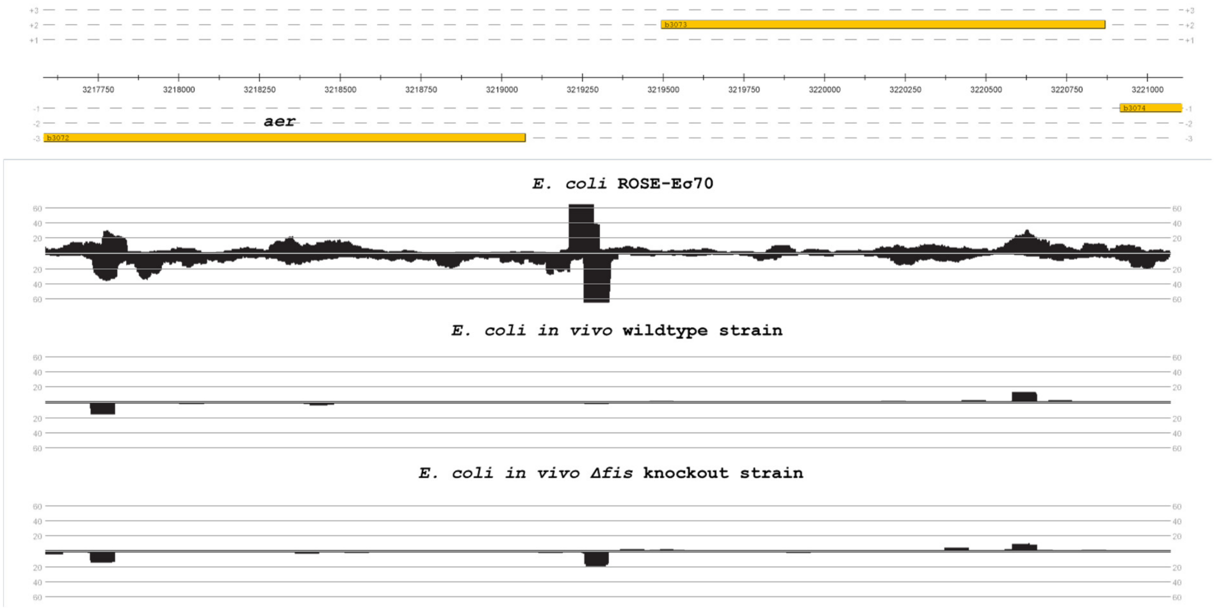

Figure S8. Coverage of mapped reads on the reference genome with an emphasis on gene *aer*. Readcount in the promoter region of *aer* from the *E. coli* ROSE-Eσ^{70} (top), *E. coli in vivo* Wildtype strain (middle) and *E. coli in vivo* Δ*fis* knockout strain (bottom). The mapping took place on the respective reference genome (U00096.3) and is visualized with ReadXplorer [13].
